# Salt–Alkali Gradient Correlates with Distinct Bacterial Communities of *Salicornia europaea* L. Across Soil–Root–Leaf Compartments in Guhya Salt Lake

**DOI:** 10.3390/microorganisms14071577

**Published:** 2026-07-20

**Authors:** Chaobing Luo, Xiu Zhang, Chenbo Tan, Yueting Lang, Hongyan Ma, Zhaojun Liu

**Affiliations:** 1 Biological Breeding Laboratory, Xinjiang Uygur Autonomous Region Academy of Agricultural Sciences, Urumqi 830091, China; 13366181512@163.com (C.L.); zhangxiuqwer1234@163.com (X.Z.); tcbdeyouxiang@gmail.com (C.T.); 17723269510@163.com (Y.L.); mahongyan@xaas.ac.cn (H.M.); 2 Xinjiang Key Laboratory of Crop Biotechnology, Xinjiang Uygur Autonomous Region Academy of Agricultural Sciences, Urumqi 830091, China; 3 College of Horticulture Science, Zhejiang A&F University, Hangzhou 311300, China; 4 Xinjiang Institute of Ecology and Geography, Chinese Academy of Sciences, Urumqi 830011, China

**Keywords:** salt–alkali gradient, halophytic plant, bacterial community structure, biomarkers

## Abstract

Bacteria play a vital role in enhancing host resistance to salt–alkali stress. However, the composition and structure of bacterial communities associated with halophytes under natural high salt–alkali conditions remain poorly understood. Here, a transect comprising six sampling points (0, 9, 18, 27, 36, and 45 m) was established across a natural population of *Salicornia europaea* L. in Guhya Salt Lake salt–alkali soils. At each sampling site, bulk soil, rhizosphere soil, root, and leaf samples were collected for 16S rRNA gene amplicon sequencing. The pH and soil electrical conductivity (EC) significantly increased along the sampling sites establishing a distinct salt–alkali gradient. This gradient provides an ideal model for studying the ecological adaptation mechanisms of halophytes and their related microorganisms. The results showed that alpha diversity (Shannon index) of bacterial communities significantly decreased across sampling sites in bulk soil, rhizosphere soil and root, but not in leaf. Beta diversity varied significantly across sampling points in all sample types examined. Linear Discriminant Analysis Effect Size (LEfSe) identified specific microbial biomarkers (such as *Halomonas* spp.) for each sampling point and sample type, many of which are known salt–alkali-tolerant lineages. Random forest and correlation analysis indicated that soil chemical properties had a clear impact on these identified biomarkers. Overall, salt–alkali gradient was associated with habitat-specific microbial communities across plant compartments and certain bacterial taxa were found to be enriched in specific niches. These taxa include known salt–alkali-tolerant lineages, and may putatively contribute to host adaptation to extreme environments, which provides deeper insights into plant–microbe interactions in natural ecosystems and offers potential microbial resources for improving crop salt–alkali tolerance.

## 1. Introduction

Salt–alkali stress, characterized by the concurrent elevation of soluble salts and high pH, constitutes a pressing global environmental challenge, which affects crop performance, soil health and ecosystem functioning, arising from either natural factors or human-induced activities [1,2]. Interestingly, in natural salt–alkali ecosystems, certain halophytic plants survive and thrive, demonstrating remarkable adaptations to high salinity and alkalinity [3]. However, sophisticated adaptive strategies of halophytes to salt–alkali conditions, particularly those mediated by their associated microbiomes, remain inadequately characterized, and their potential applications in sustainable agriculture and land rehabilitation are yet to be fully realized.

Microorganisms play essential roles in sustaining ecosystem functioning, facilitating plant growth, and enhancing host resilience to abiotic stresses [4,5,6]. Studies have demonstrated that microbial communities can be substantially reshaped in salt–alkali environments [7,8], and these communities, in turn, contribute to plant salt–alkali tolerance through multiple mechanisms [9,10]. These mechanisms include phytohormone modulation, enhanced nutrient acquisition, maintenance of ionic balance, reinforcement of antioxidant defenses, and stimulation of osmolyte accumulation [11]. Consequently, systematic investigation of the dynamics of microbial communities along natural salt–alkali gradients is critical for advancing our understanding of microbe–plant–salt–alkali interactions and for developing effective microbiome-based strategies to mitigate salt–alkali stress. Notably, under extreme salt–alkali conditions, host plants can actively recruit beneficial microbial taxa from the surrounding soil, a process known as the “cry for help” strategy, which may be critical for host survival [12,13,14].

Soil chemical properties, including pH, organic matter, soil nutrients, and available nutrients, are also key factors driving the structure, diversity, and function of microbial communities [15,16,17]. In high-salinity and high-alkalinity environments, changes in soil chemical properties may reduce microbial diversity, while also selecting haloalkalitolerant taxa, including specific representatives of the Actinobacteria and Firmicutes phyla [18,19]. These environmentally selected haloalkalitolerant microbes play essential roles in salt–alkali soil ecosystems through their involvement in nutrient cycling, synthesis of osmoprotectants, and enhancement of plant resilience to stress [20].

In the plant–microbe interaction system, distinct microbial communities colonize different compartments such as the rhizosphere, root system, and aboveground tissues [21,22,23]. *Salicornia europaea* L. is a model halophyte that thrives in coastal and inland saline habitats; however, the diversity and composition of its bacterial communities across different plant compartments under natural salt–alkali gradients remain poorly understood. In this study, we established a transect comprising six sampling points (0, 9, 18, 27, 36, and 45 m) across a natural population of *S. europaea* L. in Guhya Salt Lake, where a distinct salt–alkali gradient was confirmed by increasing electrical conductivity (EC) values and pH. We hypothesized (1) the spatial salt–alkali gradient would drive contrasting assemblies across plant compartments; (2) specific salt–alkali-tolerant bacterial taxa would be enriched across plant compartments; and (3) soil chemical properties would serve as key environmental filters shaping these compartment-specific microbial communities. To test these hypotheses, bacterial communities of *S. europaea* L. in bulk soil, rhizosphere soil, root and leaf were analyzed to elucidate Salicornia’s microbial community structure in saline–alkaline environments, which provided a theoretical foundation and microbial resources for leveraging beneficial microbes to improve salt tolerance in crops.

## 2. Materials and Methods

### 2.1. Site Description and Sampling

Samples were collected from the Guhya Salt Lake (43.38° N, 88.13° E), located in the arid Dabancheng area of Urumqi, Xinjiang, China. The natural salt–alkali gradient here forms a unique natural laboratory to investigate how varying salt–alkali conditions shapes microbial communities. *S europaea* L., a typical halophyte, colonizes the salt–alkali soils surrounding the Guhya Salt Lake. Six sampling sites (0, 9, 18, 27, 36, and 45 m) were established along an *S. europaea* L. distribution transect. The starting point (0 m) was set at the landward boundary of the *S. europaea* L. population, with successive sampling zones established at 9 m intervals. Within each zone, five replicate sampling points were selected, each containing six individual plants, and four sample types (bulk soil, rhizosphere soil, root, and leaf) were collected from each point. Bulk soil samples were collected from areas at least 3 cm away from any visible roots. After gently removing the loosely adherent bulk soil, the rhizosphere soil samples (tightly adhering to the roots) were carefully collected by scraping with a sterile blade. Both bulk and rhizosphere soils were sieved through a 2 mm mesh. The roots and leaves were cleaned with sterile water, then surface-disinfected with 70% ethanol and 2% NaClO, and sonicated to minimize surface microorganisms [24,25]. All samples were stored at −80 °C until DNA extraction.

### 2.2. Soil Chemical Properties Determination

The concentrations of total nitrogen, total phosphorus, total potassium, available nitrogen, available phosphorus, available potassium and organic matter; pH; and EC (electrical conductivity) were measured according to Dong et al. (2024) with modifications [26]. In brief, the concentration of total nitrogen was measured using the Kjeldahl digestion method. The total phosphorus and total potassium contents were determined by NaOH Fusion Methods. Available nitrogen was determined by the alkaline hydrolysis diffusion method. Available phosphorus was analyzed by the molybdenum blue spectrophotometric method. Available potassium was measured using the ammonium acetate (NH4OAc) extraction method. And soil organic matter content was quantified by the potassium dichromate oxidation volumetric method. The pH and EC were measured in a mixture with a soil:water ratio of 1:5 (*w*/*v*) using a pH meter (PHSJ-6L, Leici, Shanghai, China) and an EC meter (DDSJ-308A, Leici, Shanghai, China), respectively.

### 2.3. DNA Extraction and Illumina Miseq Sequencing

Genomic DNA extraction from soils (bulk soil and rhizosphere soil) and tissues (root and leaf) was performed using the E.Z.N.A. Soil/Tissue DNA Kit (Omega Bio-tek, Norcross, GA, USA) according to the manufacturer’s protocol. The quality of DNA was evaluated by agarose gel electrophoresis and a NanoDrop 2000 spectrophotometer (ThermoFisher Scientific, Wilmington, DE, USA). Amplification of the V5-V6 regions of the 16S rRNA gene in all samples was performed using the primers 799F (5′-AACMGGATTAGATACCCKG-3′) and 1115R (5′-AGGGTTGCGCTCGTTG-3′) to exclude chloroplast DNA [27], which were modified with unique eight-base barcodes at their 5′ ends for each sample. PCR reactions were carried out in triplicate 20 μL mixtures containing 4 μL of 5 × FastPfu Buffer, 2 μL of 2.5 mM dNTPs, 5 μM of each primer, 0.4 μL of FastPfu Polymerase, and 10 ng of template DNA. The PCR amplification products of each sample were identified and purified, and then sequenced on the Illumina Miseq platform using 2 × 300 bp paired-end sequencing. Raw sequence reads were performed as per the description of Jin et al. [28]. Briefly, the raw data was split, assembled, and processed using QIIME 2 (http://qiime.org/, accessed on 10 September 2025), and clustered at 97% similarity for operational taxonomic units (OTUs) with USEARCH (version 6.1.544). Then, the OTUs of the bacteria were classified through the SILVA 138.2 (https://www.arb-silva.de/, accessed on 10 September 2025) databases. Illumina Miseq sequencing generated 4,061,605 high-quality bacterial sequences with an average read length of 300 bp. All samples exhibited excellent coverage (>96%), indicating comprehensive capture of microbial diversity.

### 2.4. Data Analysis

Statistical analyses were performed using R software (version 4.4.1) (http://www.r-project.org/, accessed on 10 September 2025). For each sample type (bulk soil, rhizosphere soil, root, and leaf), one-way ANOVA was performed to analyze the differences in soil chemical properties, microbial alpha diversity and relative abundance of the top 10 most abundant phyla across sampling sites at *p* < 0.05. Bacterial alpha diversities were calculated as the Shannon index using the “vegan” (version 2.6-6) package. Beta diversity was visualized at the OTU level using Principal Coordinate Analysis (PCoA) based on Bray–Curtis in the “vegan” and “ggplot2” (version 4.0.2) packages, and permutational multivariate analysis of variance (PERMANOVA) was performed to test the differences in community dissimilarity across sampling sites using the “vegan” package. Linear Discriminant Analysis Effect Size (LEfSe) (version 1.22.0) was performed to identify significant differences in the abundance of bacterial taxa (biomarkers) across sampling sites using the “microeco” (version 2.0.0) package [29]. Random forest analysis was performed to assess the contribution of soil chemical properties to core microbes using the “randomForest” (version 4.7-1.2) package. To understand the co-occurrence pattern among OTUs, a correlation matrix was used by calculating all pairwise Spearman’s rank correlations using the “psych” (version 2.5.3) package. The visualization and calculation of important topological features of co-occurrent networks were performed using Gephi software (version 0.9.2).

## 3. Results

### 3.1. Soil Chemical Properties

**Figure 1:**
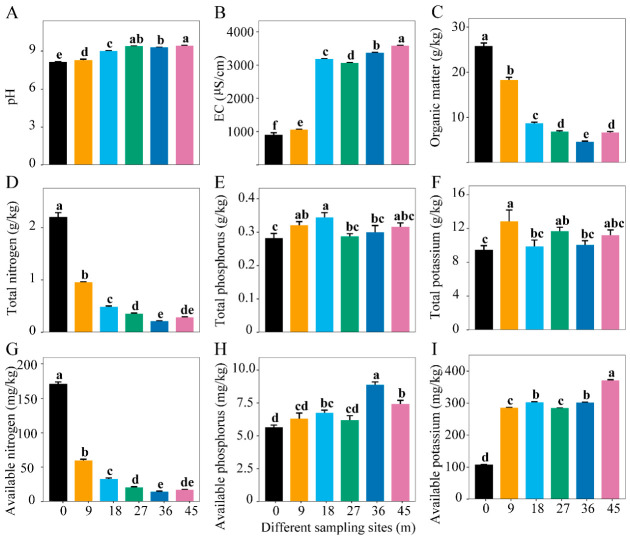
Soil chemical properties across different sampling sites. Different lowercase letters represent significant differences (*p* < 0.05). EC: electrical conductivity. (**A**) Soil pH; (**B**) EC; (**C**) organic matter; (**D**) total nitrogen; (**E**) total phosphorus; (**F**) total potassium; (**G**) available nitrogen; (**H**) available phosphorus; (**I**) available potassium.

All measured soil chemical properties showed clear tendencies across the selected sampling sites (Figure 1). While the value of pH steadily increased from about 8 to over 9, organic matter sharply reduced by about 5 times (Figure 1A,B). In contrast, EC exhibited a substantial rise from around 1000 to 3000 µS/cm (Figure 1C). To further understand major nutrient availability among the sampling sites, we quantified total and available nitrogen, phosphorus and potassium. While available phosphorus and potassium exhibited an overall increasing trend from 0 to 45 m, total and available nitrogen decreased along the sampling sites, suggesting different mechanisms for these three nutrients (Figure 1D–I). Taken together, our data showed a saline–alkaline and nutrient availability gradient window within our sampling sites, which provides an ideal system for investigating the ecological adaptation mechanisms of halophytes and their associated microorganisms.

### 3.2. Bacterial Community Structure and Composition Across Different Sampling Sites

**Figure 2:**
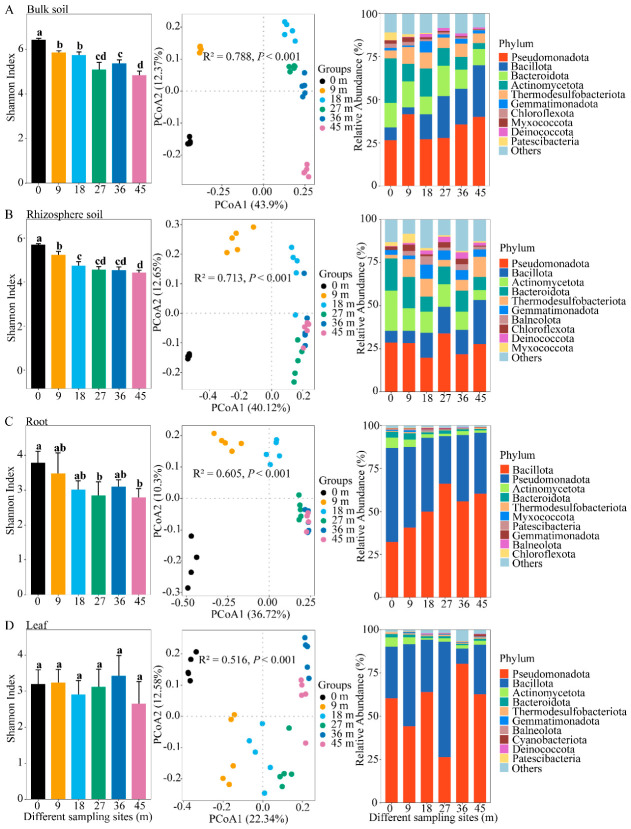
Bacterial community structure and composition across different sampling sites. Bacterial alpha diversity (Shannon index), beta diversity (PCoA ordination based on the Bray–Curtis dissimilarity matrix at the OTU level), and percent relative abundance of the top 10 most abundant phyla of (**A**) bulk soil, (**B**) rhizosphere soil, (**C**) root and (**D**) leaf. Different lowercase letters represent significant differences (*p* < 0.05).

In bulk soil, the alpha diversity (Shannon index) exhibited a declining trend along the sampling sites (Figure 2A). PCoA analysis clearly separated bulk soil samples among six sampling sites, suggesting different sampling sites had a strong influence on microorganisms of bulk soil (Figure 2A, Table S1). At the phylum level, the predominant bacterial communities consisted of Pseudomonadota, Bacillota, Bacteroidota, Actinomycetota, and Thermodesulfobacteriota (Figure 2A). The relative abundance of Bacillota generally increased from 0 to 45 m (Figure S1). In contrast, the relative abundances of Chloroflexota, Myxococcota, and Patescibacteria decreased along the gradient (Figure S1).

Similarly to bulk soil, alpha diversity of the rhizosphere soil also gradually decreased from 0 to 45 m (Figure 2B). Significant shifts in beta diversity were likewise observed among the different sampling bands (Figure 2B, Table S1). The dominant bacterial phyla identified included Pseudomonadota, Bacillota, Actinomycetota, Bacteroidota, Thermodesulfobacteriota and Gemmatimonadota (Figure 2B). Notably, the relative abundances of Bacillota and Deinococcota tended to increase from 0 to 45 m (Figure S2). Conversely, a declining trend was detected in the relative abundance of Actinomycetota along the gradient (Figure S2).

In the root, a significant decrease in alpha diversity (Shannon index) was observed from 0 to 45 m (Figure 2C). Root microorganisms showed clear differences at 0, 9, 18, 27, 36 and 45 m according to PCoA analysis, suggesting the plant root might maintain a stable micro-environment for the microorganisms (Figure 2C, Table S1). The bacterial community was predominantly composed of Bacillota and Pseudomonadota, which together accounted for over 90% of the mean relative abundance (Figure 2C). A general increasing trend was observed in the relative abundance of Bacillota (Figure S3). In contrast, Pseudomonadota and Actinomycetota displayed a decreasing trend in abundance along the spatial gradient (Figure S3).

In the leaf, no significance was observed in alpha diversity (Shannon index) across sampling sites (Figure 2D). Beta diversity showed significant differences across the sampling bands (Figure 2D, Table S1). Pseudomonadota and Bacillota were the dominant phyla, which accounted for more than 90% of the sequences (Figure 2D, Figure S4).

### 3.3. Soil Chemical Properties Shaping the Biomarkers in Various Sample Types

**Figure 3:**
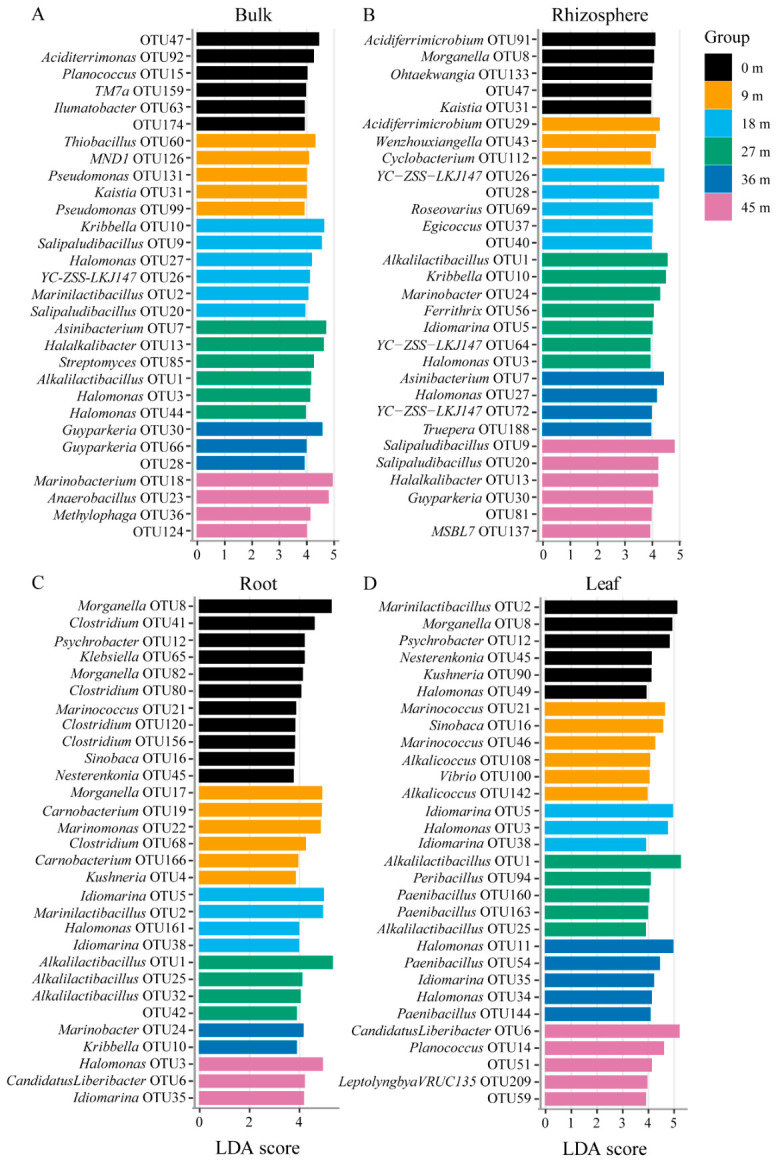
Top 30 biomarkers identified by LEfSe (LDA score > 2) in (**A**) bulk soil, (**B**) rhizosphere soil, (**C**) root and (**D**) leaf. Taxa that could be assigned to the genus level were shown as genus, otherwise they were shown as OTU id.

LEfSe analysis identified 959, 992, 286, and 249 differential OTUs along the sampling sites in bulk soil, rhizosphere soil, root, and leaf, respectively. The top 30 OTUs based on LDA scores were selected as key biomarkers, potentially serving as crucial microorganisms that facilitate growth of *S. europaea* L. In bulk soil, biomarker distribution showed 6, 5, 6, 6, 3, and 4 OTUs specifically enriched at sites of 0, 9, 18, 27, 36, and 45 m, respectively. These predominant biomarkers were primarily affiliated with the genera *Halomonas*, *Pseudomonas*, *Salipaludibacillus*, and *Guyparkeria* (Figure 3A). In rhizosphere soil, five, three, five, seven, four, and six biomarkers were enriched at the corresponding sites, predominantly from the genera *Acidiferrimicrobium*, *Halomonas*, and *Salipaludibacillus* (Figure 3B). In the root, the distribution of biomarkers was 11, 6, 4, 4, 2, and 3 OTUs, mainly representing *Clostridium*, *Morganella*, *Idiomarina*, *Alkalilactibacillus*, *Carnobacterium*, and *Halomonas* (Figure 3C). In the leaf, six, six, three, five, five, and five biomarkers were identified, largely belonging to *Halomonas*, *Paenibacillus*, *Alkalilactibacillus*, *Idiomarina*, *Marinococcus*, and *Alkalicoccus* (Figure 3D).

**Figure 4:**
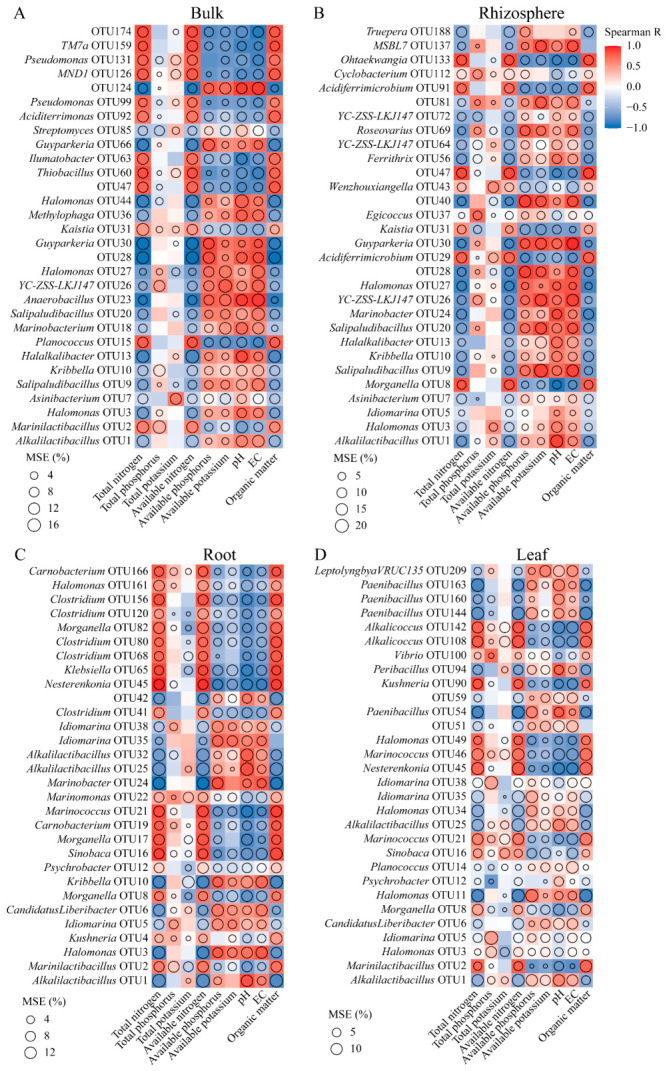
Random forest analysis showed the contribution of soil chemical properties to key biomarkers in (**A**) bulk soil, (**B**) rhizosphere soil, (**C**) root and (**D**) leaf. The size of the circle represents the importance of each variable (% increase in MSE). The color gradient represents Spearman’s correlation between the relative abundance of each OTU and each soil chemical property. Taxa that could be assigned to the genus level were shown as genus, otherwise they were shown as OTU id. EC: electrical conductivity.

Random forest analysis showed that soil chemical properties had clear influences on biomarkers in all sample types, with distinct patterns observed in bulk soil, rhizosphere soil, root, and leaf (Figure 4). The importance of each chemical variable, as measured by the percentage increase in mean squared error (% IncMSE), varied considerably among different microbial taxa. Spearman correlation analysis further revealed positive and negative associations between specific soil properties and the individual OTUs, reflecting habitat-specific microbial responses to environmental factors. The effects of total soil phosphorus and potassium on biomarkers were relatively minor compared with other factors.

### 3.4. Co-Occurrence Networks of the Bacterial Community

**Figure 5:**
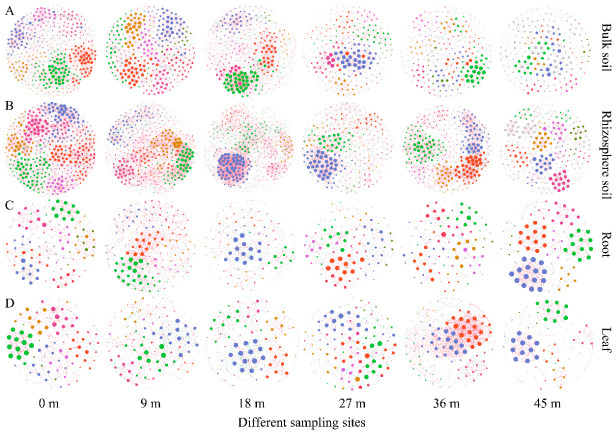
The co-occurrence networks showed the associations of microbial communities at the OTU level (relative abundance >0.05%) across sampling sites in (**A**) bulk soil, (**B**) rhizosphere soil, (**C**) root and (**D**) leaf. The pink connection between two nodes represents a significantly positive correlation (R > 0.6; *p* < 0.01), and blue represents a significantly negative (R < 0.6; *p* < 0.01) correlation. The node size is proportional to the number of connections and node color represents the different modules.

The co-occurrence networks showed different patterns of bacterial communities across sampling sites (Figure 5, Table S2). In the bulk soil, the number of nodes (from 359 to 207) and edges (from 3670 to 722), average degree (from 20.45 to 6.98), and percent of positive edges (from 93.11% to 68.56%) generally decreased along the sampling sites. Average path length (from 4.96 to 6.55) and average clustering coefficient (from 0.670 to 0.856) gradually increased along the sampling sites. The modularity initially remained stable but showed a substantial increase at the highest salt–alkali level (from 0.674 to 0.800) (Figure 5A, Table S2).

In the rhizosphere soil, network size (nodes and edges) also decreased with sampling site. The average degree declined from 15.81 to 6.74. The percent of positive edges plummeted from 92.50% to 66.99%. The modularity first decreased from 0.719 (0 m) to 0.574 (18 m), and then increased to 0.880 at 45 m (Figure 5B, Table S2).

In the root, the network patterns of bacterial communities showed a non-linear response to increasing salinity and alkalinity. The average path length peaked at 7.835 (36 m) before crashing to 1.000 at 45 m. Simultaneously, the average clustering coefficient reached its maximum value of 1.000, and the average degree increased to 7.457. The modularity remained high throughout but peaked (0.814) at 45 m. A decline was observed in the percent of positive correlations, which decreased from 87.05% to 60.64% (Figure 5C, Table S2).

In the leaf, a unique response pattern was observed. The average path length generally decreased (from 6.49 to 1.19). However, the percent of positive edges in the leaf did not show a consistent decline. It dropped initially but then rebounded to 90.21% at the highest salt–alkali level (Figure 5D, Table S2).

## 4. Discussion

Understanding the composition of and driving factors for microbial communities in halophyte habitats along salt–alkali gradients is essential to decipher the ecological adaptation strategies employed by halophytes [30]. In this study, *S. europaea* L. populations were selected as the research subject. Six representative sampling zones were established along the species’ ecological distribution gradient. Soil pH and electrical conductivity (EC) measurements showed significant differences among the sites (*p* < 0.05), confirming a distinct natural salt–alkali gradient across the sampling sites. This environmentally graded system provided a valuable natural laboratory for examining how salt–alkali heterogeneity influences the structure of halophyte-associated microbial communities.

Soil salinity and alkalinity act as environmental filters, excluding intolerant microbial taxa and thereby driving significant changes in microbial diversity [18,31]. Our results demonstrated a significant decline in bacterial alpha diversity (Shannon index) across the salt–alkali gradient in the bulk soil, rhizosphere, and root compartments, which is consistent with previous research findings. In contrast, the leaf-associated microbial community exhibited no significant response to increasing salinity levels. This pattern is likely generated because the leaf-associated microbiota is predominantly shaped by atmospheric conditions and plant genetic factors, rather than direct soil properties [32,33]. Similar decoupling between above- and belowground microbial communities has been documented in other ecosystems, highlighting their divergent responses to environmental drivers [34]. Furthermore, we observed significant differences in β-diversity across the sampling sites in all sample types, suggesting that each sample may be enriched with a distinct set of key microbial taxa [35].

Soil salinity and alkalinity can also modify the composition of bacterial communities. Across all examined compartments (bulk soil, rhizosphere soil, root, and leaf), the bacterial communities were dominated by a few core phyla, primarily Pseudomonadota and Bacillota. This low complexity is a common feature in high-stress environments, where a limited number of tolerant taxa outcompete others [36]. The increase in the relative abundance of Bacillota across bulk soil, rhizosphere soil, and root compartments indicated their selective advantage under salt–alkali conditions, which may be attributed to their stable cell membrane structure and rapid response to oxidative stress [37,38]. The observed decline of Actinomycetota and Chloroflexota along the gradient suggested their relative sensitivity to salt–alkali stress. While known for their metabolic versatility in soil, many Actinobacteria may be outcompeted by more specialized haloalkalitolerant organisms under extreme conditions [18,39,40]. Similarly, Chloroflexota, often involved in anaerobic processes, might see their niche space reduced due to salt–alkali-induced changes in soil physicochemical properties [41,42]. These phylum-level changes provided clear evidence of microbial compositional responses to the salt–alkali gradient.

To identify key microbial taxa associated with salt–alkali tolerance, we performed LEfSe (Linear Discriminant Analysis Effect Size) analysis to detect statistically significant biomarkers. The distinct number and distribution of biomarkers across the sampling sites within each compartment indicated that salt–alkali was associated with a finely structured ecological landscape, selecting for unique microbial assemblages. The recurrent identification of *Halomonas* across bulk soil, rhizosphere soil, root, and leaf compartments highlights its remarkable ecological adaptability and prevalence in salt–alkali environments. As a widely reported halophilic genus, *Halomonas* is known for its ability to synthesize compatible solutes—such as ectoine and proline—to maintain cellular osmotic balance [43,44]. The enrichment of biomarker OTUs belonging to this genus is consistent with its known osmoprotective capacity, although their direct contribution to host fitness remains to be experimentally verified. The enrichment of OTUs identified as *Clostridium* and *Morganella* is also notable. *Clostridium* species are characterized by their anaerobic fermentative metabolism, which may favor their persistence in hypoxic environments like plant roots [45]. Similarly, some *Morganella* species have been reported to stimulate plant antioxidant enzyme activity [46]. The dominance of *Paenibacillus* and *Marinococcus* in the leaf was also observed. These genera include known plant growth-promoting promoters and antagonists of fungal pathogens, and *Paenibacillus* may enhance host adaptability [47,48,49]. *Marinococcus*, as a type of halophilic bacterium, might mitigate ion imbalance and toxicity in its host plant [50,51]. Collectively, these key microbial taxa were differentially distributed across plant compartments and are largely consistent with known halotolerant lineages. However, further validation is still needed to understand their roles and functions.

Random forest and correlation analyses demonstrated that soil chemical properties serve as key regulatory factors influencing critical biomarkers, a finding that has been consistently observed across multiple studies [18,52]. The significant variation in response to chemical variables across different microbial taxa indicated that the salt–alkali condition drives niche differentiation of bacterial communities [31]. The relatively weak influence of total phosphorus and potassium is particularly noteworthy. This may suggest that the bioavailability of these elements rather than their total content plays a critical role in microbial selection. Alternatively, their effects may be overshadowed by stronger drivers such as EC or pH, which have been identified as dominant environmental filters in salt–alkali agroecosystems [18,53]. In addition, soil moisture may act as a key factor, together with other physicochemical properties, to shape microbial community structure [22]. Further studies are still needed to focus on the effects of nutrient availability and moisture on microorganisms.

The co-occurrence network exhibits significantly different patterns in different compartments. Overall, the number of nodes and edges and mean degree all decreased with increasing salt–alkali stress, indicating that more extreme conditions are associated with reduced network complexity, which is consistent with previous research results [18]. Meanwhile, the percent of positive edges decreased significantly with sampling sites, which may reflect intensified resource limitation (e.g., water, nutrients, and living space) under salt–alkali stress [54]. Furthermore, the consistently high modularity across all sampling sites, which reached its peak under the highest salt–alkali stress, suggests that only a limited number of highly specialized microbial modules may persist under extreme conditions [55]. Notably, the endophytic bacterial co-occurrence networks in the roots and leaves of *S. europaea* L. exhibited a distinct pattern compared with the soils, potentially suggesting host-associated filtering [56]. On the one hand, plants recruit specific beneficial microbial taxa through root exudates, including organic acids, sugars, and secondary metabolites [57,58]; on the other hand, host physiological traits—including tissue-specific oxygen gradients, pH, and nutrient profiles—impose additional selective filtering on microbial communities [59]. However, the complex molecular mechanisms governing microbial community assembly require further experimental validation.

This study demonstrated that extreme natural environments such as saline–alkaline soils are associated with the structure, diversity, and composition of microbial communities in bulk soil, rhizosphere soil, root, and leaf. We further identified several key microbial taxa (such as *Halomonas*, *Clostridium*, *Morganella*, *Paenibacillus*, and *Marinococcus*), which may putatively contribute to adaptation of *S. europaea* L. under salt–alkali conditions through diverse mechanisms—such as synthesizing compatible solutes, regulating ion homeostasis, enhancing antioxidant defenses, and stimulating host plant growth. These insights establish a theoretical basis for harnessing beneficial microorganisms to enhance crop salt–alkali tolerance and support the sustainable agricultural use of saline–alkali soils.

## 5. Conclusions

Increasing salinity and alkaline significantly reduced the alpha diversity of bacterial communities in the bulk soil, rhizosphere soil, and root compartments, but not in the leaf. Salt–alkali gradient also altered the structure and composition of bacterial communities. Distinct key taxa were enriched across sampling zones in different compartments, all of which were influenced by local chemical properties. Furthermore, salt–alkali stress reduced both the size and proportion of positive connections in the bacterial co-occurrence networks, while increasing their modularity. These findings provide important insights for understanding the role of haloalkalitolerant microbial communities in the adaptation of halophytes to salt–alkali environments and highlight potential applications in sustainable agriculture. However, future studies integrating metagenomics, metatranscriptomics, and culturomics are needed to elucidate the molecular mechanisms underlying host–microbe interactions under extreme conditions.

## Data Availability

The raw data supporting the conclusions of this article will be made available by the authors on request.

## Supplementary Materials

The following supporting information can be downloaded at https://www.mdpi.com/article/10.3390/microorganisms14071577/s1, Table S1. Pairwise comparison of beta diversity in bacterial communities; Table S2. Topological properties of co-occurrence networks of microbial communities at the OTU level (relative abundance > 0.05%) across sampling sites in bulk soil, rhizosphere soil, root and leaf; Figure S1. The percent relative abundance of the top 10 most abundant phyla exhibiting significant differences across sampling sites in bulk soil; Figure S2. The percent relative abundance of the top 10 most abundant phyla exhibiting significant differences across sampling sites in rhizosphere soil; Figure S3. The percent relative abundance of the top 10 most abundant phyla exhibiting significant differences across sampling sites in root; Figure S4. The percent relative abundance of the top 10 most abundant phyla exhibiting significant differences across sampling sites in leaf.
