# Supplementary material for: Salt–Alkali Gradient Correlates with Distinct Bacterial Communities of Salicornia europaea L. Across Soil–Root–Leaf Compartments in Guhya Salt Lake
Source: Microorganisms. 2026 Jul 20;14(7):1577. doi: 10.3390/microorganisms14071577 (PMC13413735; doi:10.3390/microorganisms14071577)
Supplement: Supplementary file 1 [file microorganisms-14-01577-s001.zip › microorganisms-4395450-supplementary.pdf]

Salt–Alkali Gradient Correlates with Distinct Bacterial Communities of *Salicornia europaea* L. Across Soil–Root–Leaf Compartments in Guhya Salt Lake

Chaobing Luo [1,2,†], Xiu Zhang [1,2,†], Chenbo Tan [1,3], Yueting Lang [1,4], Hongyan Ma [1,2] and Zhaojun Liu [1,2,*]

1 Biological Breeding Laboratory, Xinjiang Uygur Autonomous Region Academy of Agricultural Sciences, Urumqi 830091, China; 13366181512@163.com (C.L.); zhangxiuqwer1234@163.com (X.Z.); tcbdeyouxiang@gmail.com (C.T.); 17723269510@163.com (Y.L.); mahongyan@xaas.ac.cn (H.M.)

2 Xinjiang Key Laboratory of Crop Biotechnology, Xinjiang Uygur Autonomous Region Academy of Agricultural Sciences, Urumqi 830091, China

3 College of Horticulture Science, Zhejiang A&F University, Hangzhou 311300, China

4 Xinjiang Institute of Ecology and Geography, Chinese Academy of Sciences, Urumqi 830011, China

* Correspondence: lzjdeyouxiang@126.com

† These authors contributed equally to this work.

Table S1 Pairwise comparison of beta diversity in bacterial community.

	Bulk soil		Rhizosphere soil		Root		Leaf	
Group	R^2	*p*	R^2	*p*	R^2	*p*	R^2	*p*
0 m vs 9 m	0.659	0.006	0.626	0.008	0.410	0.006	0.361	0.006
0 m vs 18 m	0.757	0.005	0.738	0.010	0.573	0.015	0.412	0.006
0 m vs 27 m	0.720	0.011	0.789	0.007	0.650	0.005	0.469	0.008
0 m vs 36 m	0.766	0.008	0.793	0.007	0.679	0.011	0.553	0.007
0 m vs 45 m	0.859	0.010	0.781	0.007	0.687	0.036	0.477	0.012
9 m vs 18 m	0.729	0.008	0.480	0.008	0.363	0.009	0.267	0.005
9 m vs 27 m	0.690	0.011	0.580	0.009	0.465	0.008	0.345	0.007
9 m vs 36 m	0.743	0.010	0.585	0.007	0.505	0.016	0.488	0.014
9 m vs 45 m	0.850	0.009	0.595	0.011	0.507	0.009	0.378	0.006
18 m vs 27 m	0.454	0.006	0.452	0.011	0.338	0.010	0.271	0.010
18 m vs 36 m	0.457	0.008	0.307	0.017	0.379	0.012	0.402	0.011
18 m vs 45 m	0.679	0.009	0.382	0.007	0.389	0.004	0.328	0.006
27 m vs 36 m	0.325	0.007	0.399	0.002	0.250	0.006	0.405	0.011
27 m vs 45 m	0.588	0.011	0.476	0.011	0.281	0.007	0.260	0.020
36 m vs 45 m	0.559	0.017	0.409	0.007	0.233	0.009	0.320	0.010

Table S2 Topological properties of co-occurrence networks of microbial communities at the OTU level (relative abundance > 0.05 %) across sampling sites in bulk soil, rhizosphere soil, root and leaf.

Sample types	Sampling sites (m)	No. of nodes	No. of edges	Average degree	Average path length	Average clustering coefficient	Modularity	Percent of positive edges (%)
Bulk soil	0	359	3670	20.446	4.956	0.670	0.674	93.11
	9	303	2194	14.482	4.742	0.647	0.684	93.07
	18	299	2802	18.742	5.101	0.705	0.583	89.90
	27	230	1265	11.000	6.485	0.721	0.601	87.35
	36	247	1019	8.251	6.173	0.715	0.670	83.22
	45	207	722	6.976	6.545	0.856	0.800	68.56
Rhizosphere soil	0	384	3067	15.809	4.691	0.615	0.719	92.50
	9	333	2723	15.831	5.046	0.601	0.673	83.95
	18	318	2951	18.272	5.484	0.621	0.574	82.11
	27	259	1276	9.740	5.369	0.519	0.644	75.16
	36	284	2378	16.400	5.122	0.632	0.662	76.53
	45	242	836	6.742	5.045	0.894	0.880	66.99
Root	0	105	278	4.964	2.847	0.903	0.853	87.05
	9	142	596	8.000	6.629	0.612	0.627	85.07

	18	92	201	3.903	4.797	0.641	0.646	65.67
	27	86	215	4.3	4.495	0.538	0.671	70.23
	36	94	183	3.519	7.835	0.534	0.781	70.49
	45	84	343	7.457	1.000	1.000	0.814	60.64
Leaf	0	89	271	5.588	6.489	0.689	0.741	95.20
	9	87	270	5.567	4.135	0.639	0.652	73.70
	18	71	213	5.133	2.538	0.784	0.733	74.65
	27	98	202	3.741	8.093	0.524	0.762	68.32
	36	100	670	12.642	2.359	0.770	0.464	88.51
	45	66	143	3.531	1.190	0.973	0.759	90.21

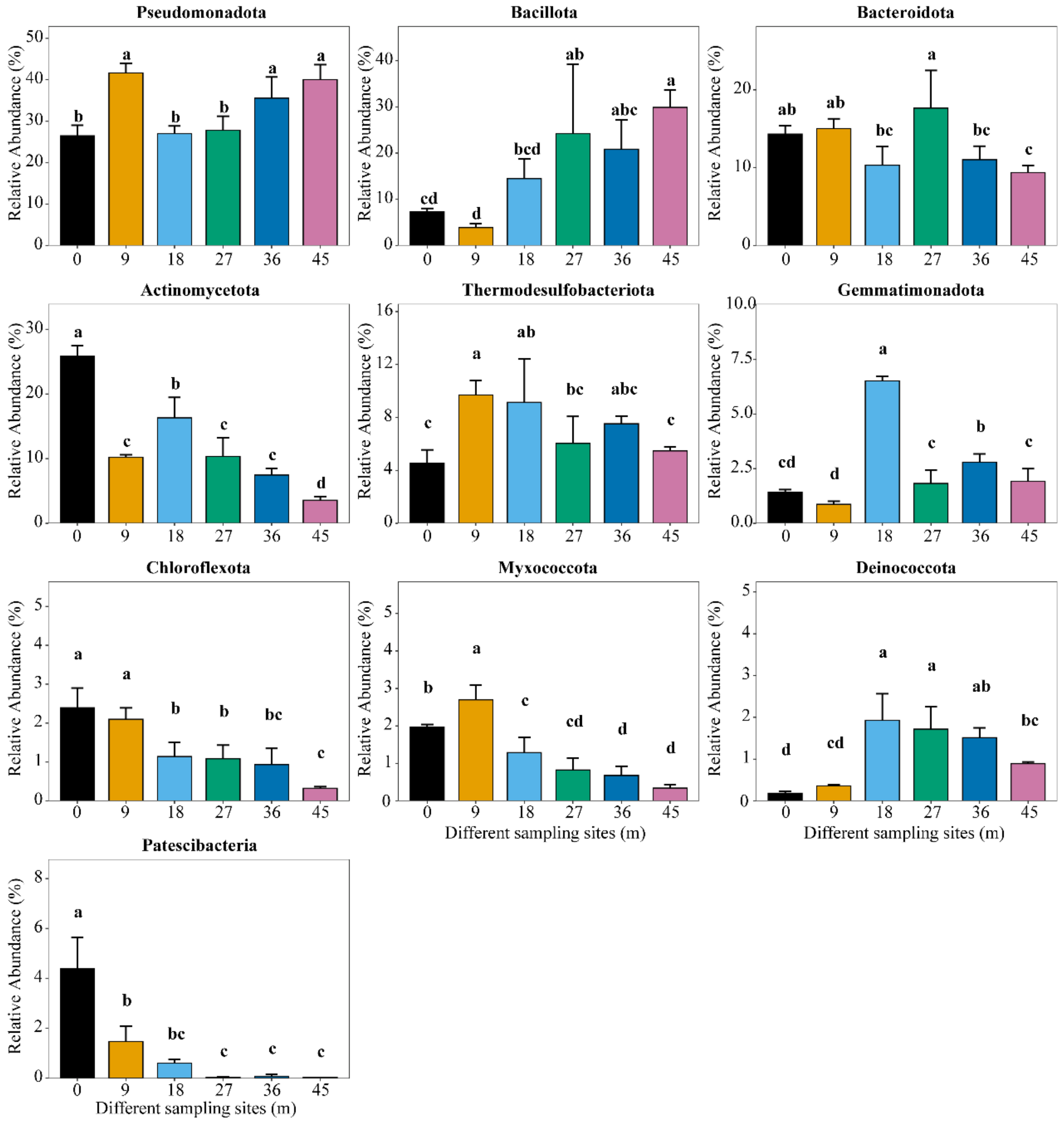

Figure S1 The percent relative abundance of the top 10 most abundant phyla exhibiting significant differences across sampling sites in bulk soil.

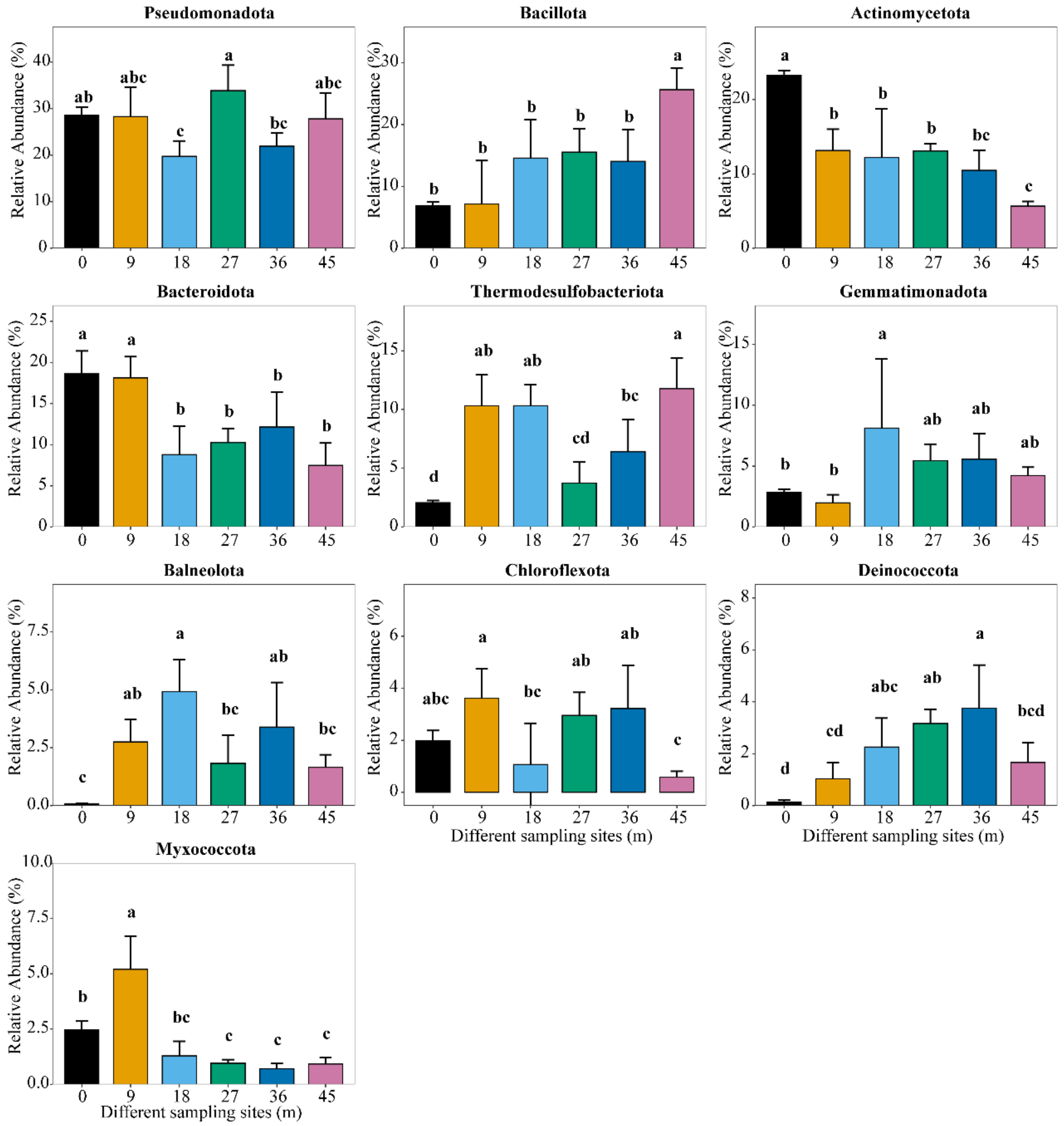

Figure S2 The percent relative abundance of the top 10 most abundant phyla exhibiting significant differences across sampling sites in rhizosphere soil.

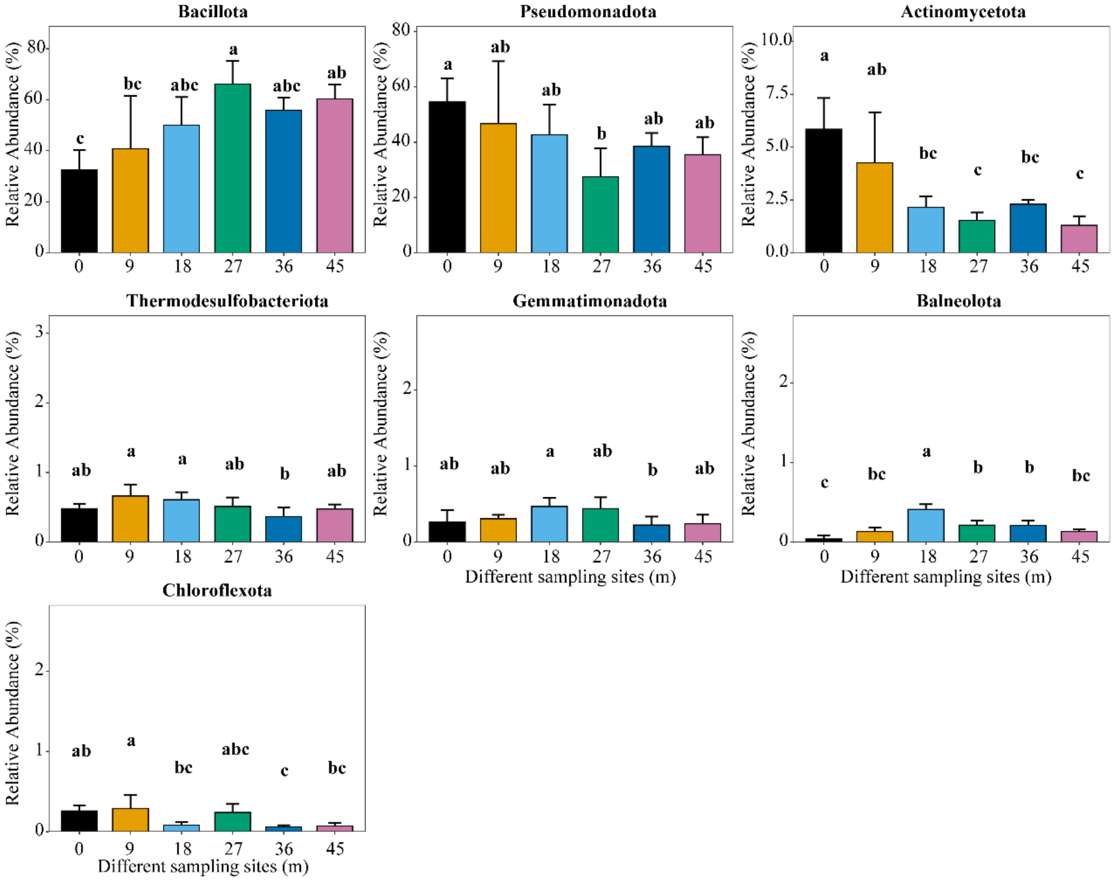

Figure S3 The percent relative abundance of the top 10 most abundant phyla exhibiting significant differences across sampling sites in root.

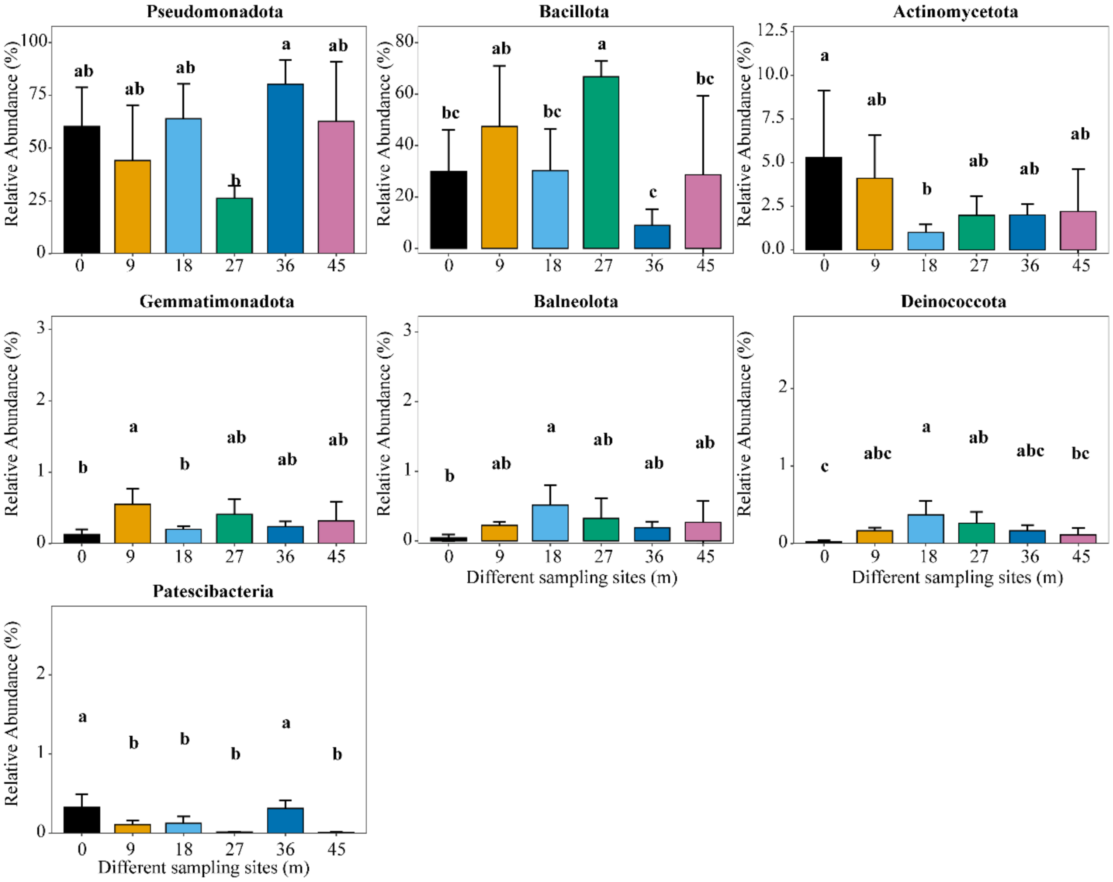

Figure S4 The percent relative abundance of the top 10 most abundant phyla exhibiting significant differences across sampling sites in leaf.
